# Improvement of Anti-Collision Performance of Concrete Columns Using Bio-Inspired Honeycomb Column Thin-Walled Structure (BHTS)

**DOI:** 10.3390/biomimetics10060355

**Published:** 2025-06-01

**Authors:** Jingbo Wang, Hongxiang Xia, Shijie Wang

**Affiliations:** 1 College of Civil and Architectural Engineering, Heilongjiang Institute of Technology, Harbin 150050, China; free-go@163.com (J.W.); wangshijie@hljit.edu.cn (S.W.); 2 School of Environment and Civil Engineering, Dongguan University of Technology, Dongguan 523808, China; 3 School of Civil Engineering, Tianjin University, Tianjin 523808, China; 4 School of Civil Engineering, Harbin Institute of Technology, Harbin 150090, China

**Keywords:** bio-inspired honeycomb structure, SLM, reinforced concrete, structural impact, numerical simulation

## Abstract

In recent years, frequent vehicle–bridge pier collision accidents have posed a serious threat to people’s economic and life security. In order to avert the impairment of reinforced concrete bridge piers (RCBPs) under the impact of vehicles, three kinds of Mg–Al alloy AlSi10Mg anti-collision structures designed by selective laser melting (SLM) printing were tested by the numerical simulation method in this study: an ultra-high performance concrete (UHPC) anti-collision structure, a bio-inspired honeycomb column thin-walled structure (BHTS) buffer interlayer, and a UHPC–BHTS composite structure were used to reduce the damage degree of RCBPs caused by vehicle impact. In accordance with the prototype configuration of the pier, a scaled model with a scale ratio of 1:10 was fabricated. Three anti-collision structures were installed on the reinforced concrete (RC) column specimens for the steel ball impact test. The impact simulation under low-energy and high-energy input was carried out successively, and the protective effect of the three anti-collision devices on the RC column was comprehensively evaluated. The outcomes demonstrate that the BHTS buffer interlayer and the UHPC–BHTS composite structure are capable of converting the shear failure of RC columns into bending failure, thereby exerting an efficacious role in safeguarding RC columns. The damage was evaluated under all impact conditions of BHTS and UHPC–BHTS composite structures, and the RC column only suffered slight damage, while the RC column without protective measures and the RC column with the UHPC anti-collision structure alone showed serious damage and collapse behavior. This approach can offer a valuable reference for anti-collision design within analogous projects.

## 1. Introduction

Honeycomb sandwich structures, due to their excellent mechanical properties of high strength and light weight [1,2,3], have been extensively utilized in the realms of civil engineering, transportation, and aerospace [4,5]. In addition, there are studies on the energy absorption (EA) of various kinds of honeycomb sandwiches [6,7], which have led some scholars to believe that honeycomb sandwiches also have excellent specific EA and are applicable in scenarios demanding impact resistance. The honeycomb sandwich structure design has been closely integrated with bionic engineering in recent years. These bionic honeycomb structures frequently possess superior mechanical properties in comparison to conventional honeycomb structures (CHS). In 2017, based on the internal structure of Coccinella septempunctata and Allomyrina dichotoma, J. Xiang and J. Du [8] proposed a new honeycomb structure with columns filled in different ways, which was named the bio-inspired honeycomb column thin-walled structure (BHTS). The researchers designed two kinds of BHTS and studied their EA characteristics under axial impact load. The outcomes demonstrate that the EA characteristics of BHTS are remarkably superior to those of CHS, so BHTS is considered to have substantial application potential within EA scenarios. In the past few years, BHTS, regarded as a novel bionic structure, has been widely recognized by researchers [9,10], and numerous investigations have been conducted on BHTS across the globe. In 2018, P. Hao and J. Du [11] studied the internal structure of the beetle Allomyrina dichotoma based on the study of Chen et al. [12], and proposed three types of BHTSs. The EA characteristics of the designed BHTSs under axial impact loading were investigated through numerical simulation. In 2023, Xia et al. [13] utilized BHTS as a substitute for an attached anti-collision structure within the realm of civil engineering. The dynamic response of the simply supported beam structure safeguarded by BHTS was investigated and expounded, and the limit displacement prediction model was presented. In 2024, H. Xia [14] undertook a theoretical investigation of the three BHTS structures proposed by P. Hao and J. Du. The mean crushing force and EA of the three BHTS structures were determined by applying the simplified superfolding unit theory. The outcomes demonstrate that the EA capacity of BHTSs is significantly greater than that of CHS, which can be widely used in impact protection scenarios.

**Figure 1:**
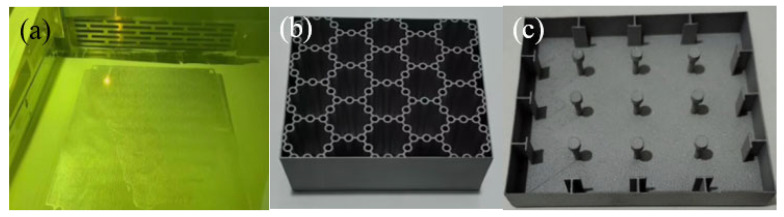
Anti-collision device made by SLM: (**a**) SLM printing process; (**b**) printed UHPC anti-collision structure panel; (**c**) printed BHTS anti-collision structure.

However, the bionic honeycomb structure often has complex geometric shapes, which bring about great difficulties to the manufacturing process. In addition, the efficiency and accuracy of traditional manufacturing technology cannot be well guaranteed. In light of this, the fabrication process of BHTS represents a suitable means to attain the practical application values of materials. Three-dimensional (3D) printing technology possesses the merits of high machining precision and a short processing cycle, enabling it to effectively fabricate complex honeycomb structures [15,16,17]. In 2022, N. San Ha et al. [18] used fused deposition modeling (FDM) printing technology to analyze the mechanical attributes and EA characteristics of the proposed bio-inspired hierarchical circular honeycomb (BHCH) and obtained good research results. The manufacturing technology has played a huge role in promoting the application of BHCH in various projects. In 2023, F. Albert et al. [11] utilized a fused filament fabrication (FFF) machine equipped with a heating chamber for the fabrication of core samples, aiming to augment layer cohesion as well as the stiffness of material joints. The research results indicated that more effective cell morphology can be achieved through additive manufacturing technology, and additive manufacturing has great potential in fabricating lightweight, high-performance sandwich panels. Selective laser melting (SLM) technology is a metal additive manufacturing method based on high-energy laser sintering. Its basic principle is to form three-dimensional solid parts by melting metal powder layer by layer. In 2024, Z. Li et al. [19,20] used the SLM, machine learning, and multi-objective optimization to achieve excellent versatility, obtain structures with excellent mechanical properties, and broaden the vision of multifunctional material design. In 2023 and 2024, Xia et al. [21,22], in order to study the material damage behavior and strain rate effect of metal materials, used SLM to prepare anti-collision structure panels composed of BHTSs and ultra-high performance concrete (UHPC). The EA characteristics of BHTS under low-velocity uniaxial compression were examined experimentally and through numerical simulation, and the EA performance of BHTS was further studied (Figure 1).

**Figure 2:**
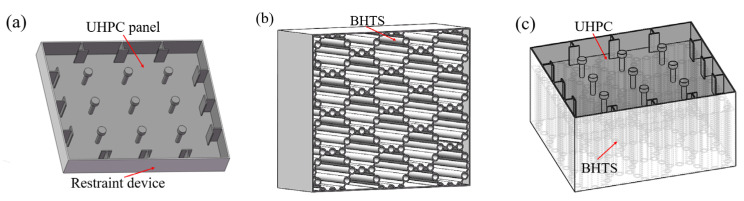
The three types of anti-collision devices are as follows: (**a**) UHPC anti-collision structure; (**b**) BHTS buffer interlayer; (**c**) UHPC–BHTS composite structure.

However, although BHTS exhibits excellent EA performance, it is prone to premature local damage, which leads to a decrease in its overall EA efficiency. Therefore, it is not suitable to be used as an EA device in isolation or applied to the field of engineering practice. In view of the increasingly serious problem of vehicle impact on bridges and the lack of research on bridge–vehicle collision protection devices, it is urgent to carry out relevant research to design several new and excellent anti-collision structures to replace traditional vehicle collision protection facilities, so as to achieve effective protection of bridge structures. At the same time, if this type of anti-collision structure can exhibit the characteristic of reusability, it would be ideal, allowing the purpose of reducing maintenance costs to be achieved. UHPC represents an ultra-high-strength cementitious material characterized by low porosity, high strength, as well as high toughness [23]. In addition, UHPC possesses favorable EA capacity and can be applied in special structures [24,25]. The impact resistance of UHPC shows a more suitable characteristic in the case of low-energy input. Independent or combined devices of UHPC and honeycomb structures are commonly used in bridge engineering to prevent vehicle–ship collisions and other collision scenarios [26]. It can act as a force-transfer panel layer and combine with BHTS, which has excellent EA capacity, to protect the BHTS in the inner layer, thereby improving the EA efficiency of the overall structure. This paper integrates the remarkable EA performance of BHTS, the high strength and toughness of UHPC, and proposes three protective structures (Figure 2): a UHPC anti-collision structure; a BHTS buffer interlayer; and a UHPC–BHTS anti-collision composite structure was set up, and the working conditions without an anti-collision structure were set as the control group. Impact tests were carried out on the above three protective structures. The purpose is to effectively avoid and alleviate the RCBP damage caused by vehicle impact, so as to fully guarantee safety during driving and reduce the degree of damage caused by accidents. At the same time, it strives to overcome a series of drawbacks of traditional protective facilities, such as poor energy consumption efficiency, lack of performance stability, and structural vulnerability. The mitigation degree of RCBP damage by the three anti-collision devices is evaluated, which furnishes a significant reference for engineers to safeguard RCBP against impact.

## 2. Experiment Methods, Materials, and Numerical Models

### 2.1. The Experimental Design of an RC Column

#### 2.1.1. Introduction of Bridge Structure

**Figure 3:**
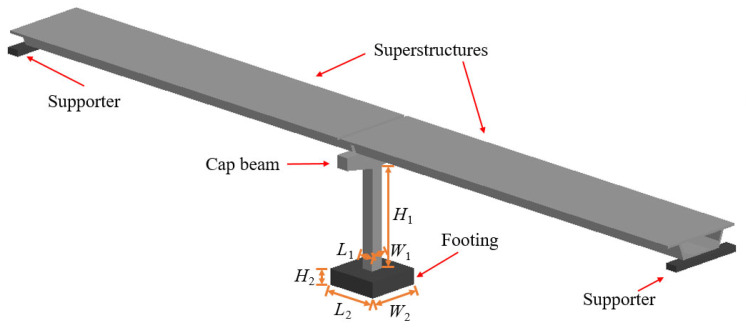
Three-dimensional view of the bridge column with superstructures.

In this research, the vehicular impact on T-type, single-pier bridges, which has been investigated by scholars both domestically and internationally, was chosen as a reference. The bridge model investigated by H. Hao et al. [27,28,29] was used to devise the model. The bridge model under consideration comprises a solitary column and a base footing, as depicted in Figure 3. The reference RCBP cross-sectional dimensions employed within this study were 1200 mm × 1200 mm (*L*_1_ × *W*_1_), with a height of 9600 mm (*H*_1_). The dimensions of the concrete footing measure 5200 mm × 5200 mm × 1500 mm (*L*_2_ × *W*_2_ × *H*_2_). The trapezoidal solid cover beam is positioned at the apex of the column for the purpose of transmitting the weight of the superstructure to the substructure. The aggregate gravitational load, which is constituted by the superstructure (approximately 4000 kN), the cover beam (267 kN), and the column itself (331 kN), amounts to around 4600 kN. The pier is reinforced by 24 longitudinal steel bars having a diameter *D*_1_ of 30 mm. The longitudinal steel bars stretch from the base footing to the cover beam. The diameter of the transverse steel bar, denoted as *D*_2_, measures 16 mm, while the spacing, designated as @_1_, amounts to 200 mm [28].

#### 2.1.2. Design of Model Pier

**Figure 4:**
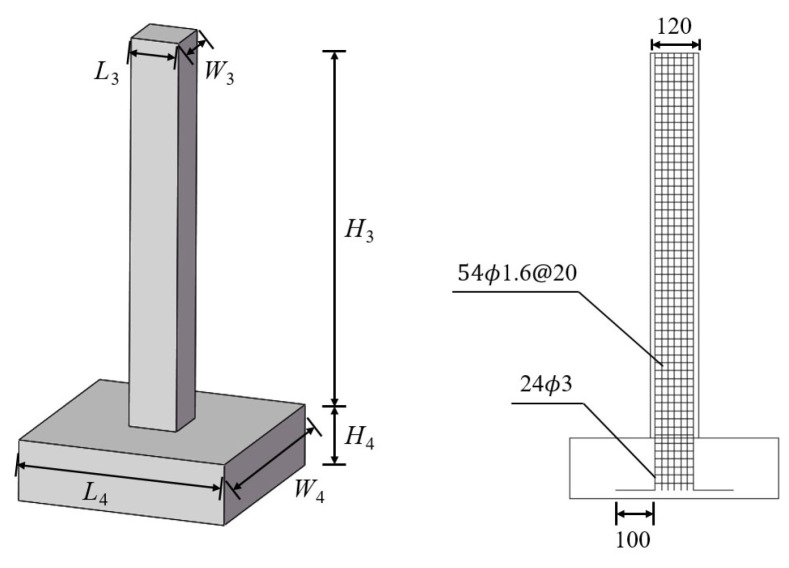
Scale model design of pier specimen.

According to the prototype pier, an RC column specimen with a model scale ratio of 1:10 was made (Figure 4). The dynamic scale model parameters acquired through the dimensional analysis approach are presented in Table 1. The elevation *H*_3_ of the scale model’s pier amounts to 960 mm, while the width *W*_3_ and the length *L*_3_ are each 120 mm. Moreover, the length *L*_4_, width *W*_4_, and elevation *H*_4_ of the pier are 520 mm, 520 mm, and 150 mm, respectively. The longitudinal reinforcing bar of the model pier uses iron wire with a diameter *D*_3_ of 3 mm. The stirrup utilizes iron wire with a diameter *D*_4_ of 1.6 mm, and the spacing @_2_ is set at 20 mm. In order to guarantee that the dynamic and static mechanical properties of the model resemble those of the prototype, it is more rational to replace the upper structure and the cover beam load with a concentrated mass.

Owing to the relatively small loading area on the pier top within the pier specimen, even upon the placement of a steel plate atop the pier, the concrete or steel block with a mass of 426.7 kg is liable to experience eccentric loading, thereby exerting an impact on the precision of the test. Consequently, the jack is utilized to exert a pressure of 4.267 kN as the axial load that is transmitted to the pier by the superstructure and the cover beam.

#### 2.1.3. Test Scheme

**Figure 5:**
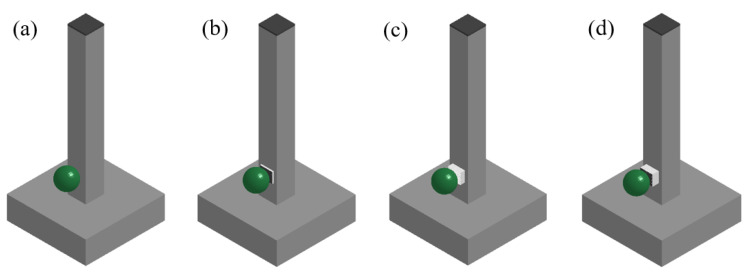
Impact test design: (**a**) an RC column; (**b**) a UHPC–RC column; (**c**) a BHTS–RC column; (**d**) a UHPC–BHTS–RC column.

Four types of columns were designed and tested: an RC column, s UHPC-RC column, s BHTS–RC column, and a UHPC–BHTS–RC column. Among them, the RC column served as the control group, lacking an anti-collision device, and the anti-collision device was mounted at an impact elevation of 150 mm, measured as a vertical distance from the foundation, as depicted in Figure 5.

### 2.2. Design of Anti-Collision Device

#### 2.2.1. UHPC Anti-Collision Structure

**Figure 6:**
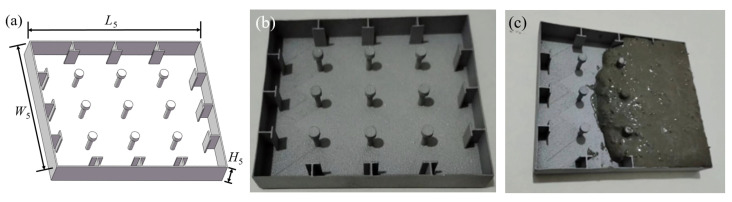
Fabrication of UHPC anti-collision structure: (**a**) geometric dimensions of restraint device; (**b**) manufacture of restraint devices; (**c**) UHPC pouring.

The UHPC anti-collision structure is composed of two parts: the restraint device and the UHPC panel. In order to improve the interfacial bonding force between the anti-collision device and the concrete, a ribbed internal constraint with a width of *W_r_* of 5 mm was designed on the inner surface of the device, as well as shear studs with a length, width, and height of *L*_6_, *W*_6_, and *H*_6_ of 80 mm, 69.28 mm, and 10 mm, respectively (Figure 6a). The device was manufactured using SLM technology, and the most commonly used silicon-containing aluminum alloy (AlSi10Mg), which has excellent mechanical properties in the SLM industry, was selected as a manufacturing sample for performance testing [30] (Figure 6b). In another independent study by the author [21], the composition and manufacturing process of the material AlSi10Mg were described in detail. Ordinary Portland cement, which exhibits a nominal compressive strength of 52.5 MPa after 28 days, is used as the primary cementitious material in UHPC. It utilizes silicon powder as an ancillary cementitious material for the enhancement of the microstructure of the hardened cement paste. Additionally, Nano-CaCO_3_ is employed to further propel and expedite the hydration process. In addition, polycarboxylate superplasticizer is incorporated into the mixture with the aim of reducing the water–cement ratio and enhancing the mechanical properties of concrete. In order to further enhance the tensile and compressive strength, as well as the toughness of UHPC, smooth steel fibers (12 mm in length, 0.2 mm in diameter) were incorporated into the UHPC mix, which was prepared according to the proportions shown in Table 1. Finally, the UHPC was filled into the device (Figure 6c).

#### 2.2.2. BHTS Buffer Interlayer

**Figure 7:**
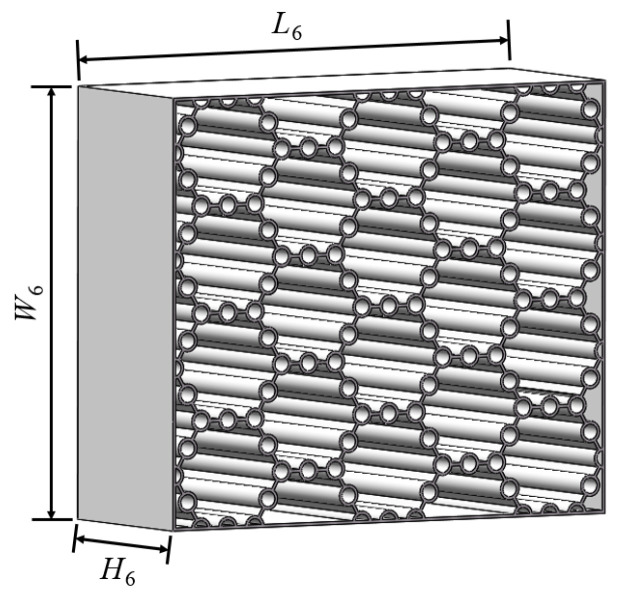
BHTS geometric dimensions.

The length, width, and height of the buffer interlayer in the BHTS, namely *L*_7_, *W*_7_, and *H*_7_, are 80 mm, 69.28 mm, and 30 mm, respectively (Figure 7). Furthermore, the manufacturing process, constituent materials, and mechanical characteristics of BHTS have been elaborated in meticulous detail in other independent investigations conducted by the author [14,21].

#### 2.2.3. UHPC–BHTS Composite Structure

**Figure 8:**
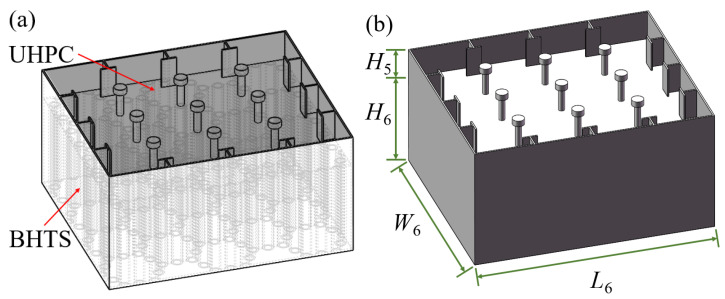
Construction of UHPC–BHTS composite structure: (**a**) schematic diagram of the UHPC–BHTS composite structure; (**b**) geometric description.

In light of the investigations conducted in Section 2.2.1 and Section 2.2.3, the UHPC–BHTS composite structure (depicted in Figure 8) was fabricated through the combination of the UHPC anti-collision structure and the BHTS buffer interlayer, and it was subsequently installed at an elevation of 150 mm from the base.

### 2.3. Numerical Model

#### 2.3.1. Numerical Model of Material and Structure

**Figure 9:**
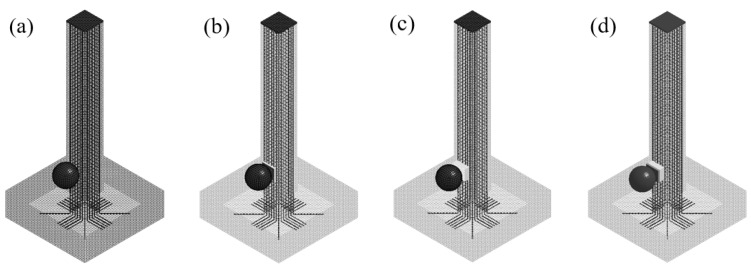
Finite element model of pier specimen: (**a**) an RC column; (**b**) a UHPC–RC column; (**c**) a BHTS–RC column; (**d**) a UHPC—HTS–RC column.

In this study, four categories of three-dimensional nonlinear finite element models regarding steel-ball impact on an RC column (Figure 9), a UHPC–RC column, a BHTS–RC column, and a UHPC–BHTS–RC column were established by using the finite element software LS-DYNA R11. The three-dimensional translational degree of freedom at the bottom of the RC column was constrained in the model; the materials of concrete, steel, UHPC, and BHTS are shown in previous independent studies [14,31,32]. Concrete columns, steel balls, and steel backing plates were depicted by hexahedral solid elements of the reduced integral constant stress type. The longitudinal reinforcements and stirrups were emulated through Hughes–Liu beam elements with 2 × 2 Gauss integration. The *MAT_CONCRETE_DAMAGE_REL3 model of concrete can simulate concrete collisions at high speeds more realistically under ultimate loads, such as impacts and explosions. It is widely used to simulate the dynamic behavior and failure mode of RC structures under impact and explosion loads [33,34]. The corresponding state equation *EOS_TABULATED_COMPACTION was employed, which is extensively utilized to simulate the dynamic behavior and failure mode of reinforced concrete structures under impact and explosion loads [33,34]. A fully integrated Belytschko–Tsay membrane element was used by BHTS, which is widely used in the static and dynamic behavior of thin-walled structures [35]. In another independent study, the constitutive model of UHPC, BHTS, and the combination structure of the two used in this paper is described in detail [14].

#### 2.3.2. Model Verification

**Figure 10:**
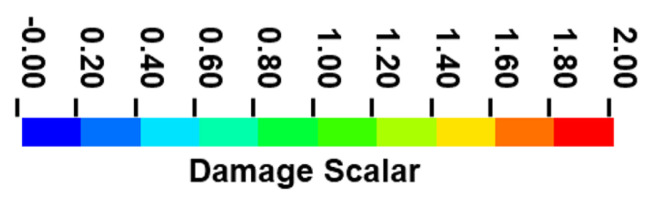
Damage Scalar.

The material mechanics model and structural verification of BHTS and UHPC have been described in previous independent studies [14,21,22], and the average error is within 5%. The numerical analysis outcomes are in high conformity with the experimental data, which effectively simulate the dynamic reaction of the test column subjected to impact loading. Consequently, it can be regarded that the present numerical model, incorporating the utilized material model and the corresponding numerical algorithm, is applicable to analyzing the dynamic characteristics of such structures under transverse impact loading. In addition, in this model, the proportional damage factor associated with the modified effective plastic strain is introduced as the damage scalar, with its variation value ranging from 0 to 2. Specifically, during the pre-peak stage, it varies within the range of 0 to 1. When it exceeds 1 and is less than 2, it mirrors the softening state of concrete. Ultimately, it approaches 2, signifying a complete failure state. The darker the color, the more severe the damage; the damage cloud diagrams of all numerical simulations in this paper are shown as the proportions in Figure 10.

## 3. Results

### 3.1. Impact Phenomenon

**Figure 11:**
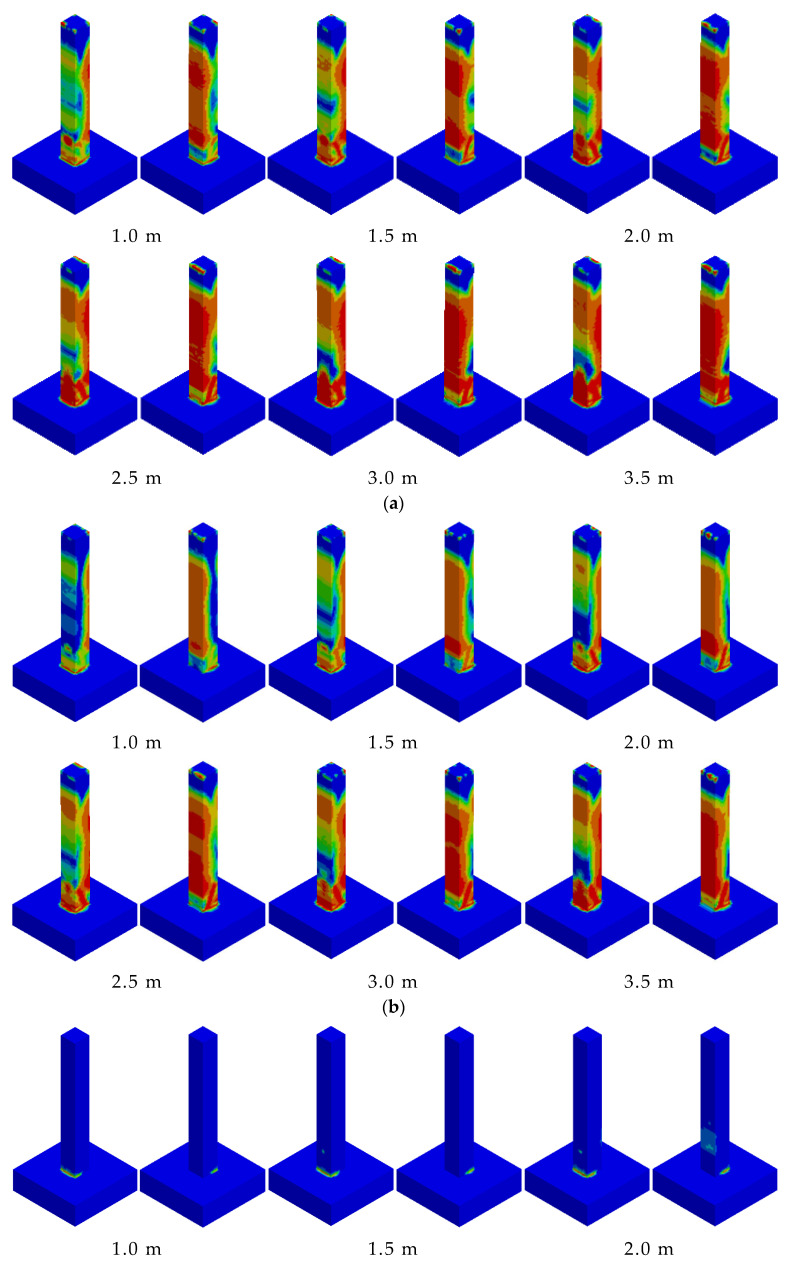

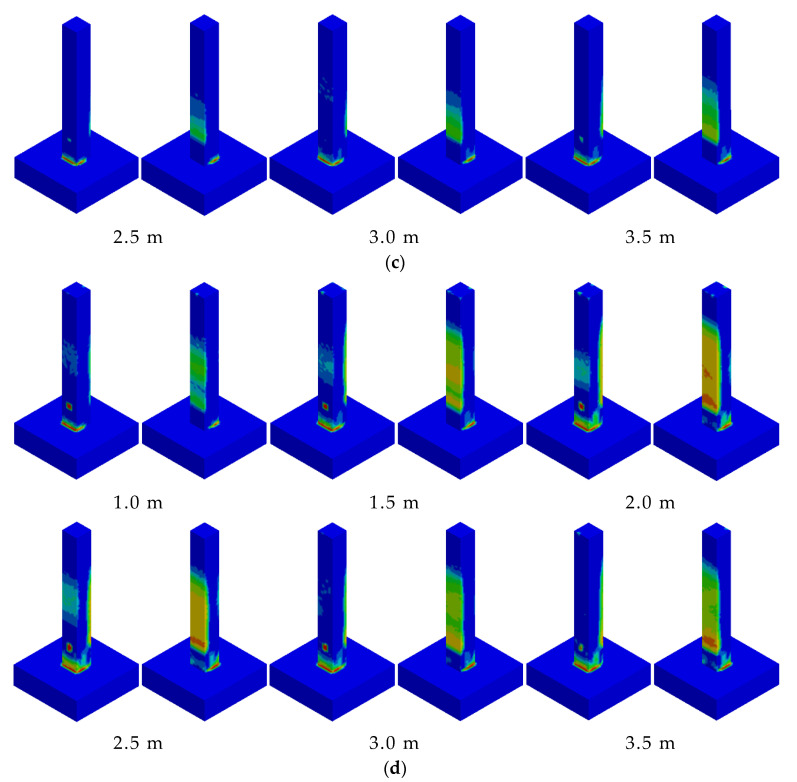
Damage comparison: (**a**) RC column; (**b**) UHPC–RC column; (**c**) BHTS–RC column; (**d**) UHPC–BHTS–RC column.

The anti-collision device was positioned at a height of 15 cm, ranging from the bottom of the RC column to the bottom of the pier. The RC column was subjected to numerical simulation tests of impact energy input without energy loss equivalent to free fall from heights of 1.0 m, 1.5 m, 2.0 m, 2.5 m, 3.0 m, and 3.5 m. The outcomes demonstrated that the RC column was severely impaired on the rear side of the impact location, and there was likewise no conspicuous shear damage. As the input energy increased, evident oblique fissures emerged in the RC column at the impact height of 1.5 m, and pronounced transverse cracks appeared on the back of the impact (Figure 11a). At the impact height ranging from 2.0 m to 3.5 m, with the augmentation of impact energy, the cracking extent of the concrete on the back of the impact was further enlarged. Under the condition of the UHPC–RC column, transverse cracks appeared on the back of the impact earlier during the impact process. With the increase in input energy, the crack was not obvious at an impact height of 1.0 m. At the impact height of 1.5 m, the RC column exhibited shear damage behavior (Figure 11b), while the RC column without an anti-collision device exhibited oblique cracks. At the impact height of 2.0 m to 3.5 m, with the increase in impact energy, the cracking extent of the concrete on the rear side of the impact was further enlarged, and conspicuous oblique fissures emerged. The situation regarding the failure of the BHTS–RC column within the range of all loading conditions, specifically from 1.0 m to 3.5 m, was markedly distinct from that of the regular RC column, as depicted in Figure 11c. Moreover, in the case of the RC column, there was no conspicuous damage manifestation. Analogous to the RC column, the plastic deformation of the RC column became more prominently evident as the loading energy was incremented.

### 3.2. Impact Force Comparison

**Figure 12:**
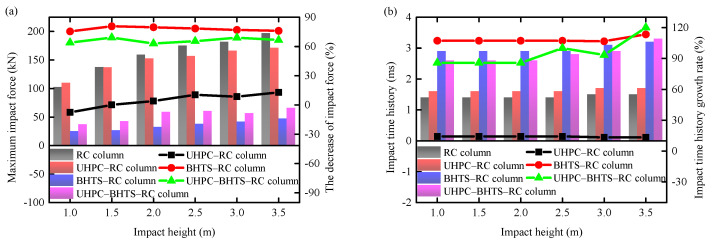
Comparison of impact force and impact time history: (**a**) force; (**b**) time history.

The impact force and impact time history of the three anti-collision devices, as well as the RC columns, are contrasted in Figure 12. Under the condition of the UHPC–RC column, the impact force of the RC column was mitigated by an average of 4.81%, and the impact time history was augmented by an average of 13.96%. Under the condition of the BHTS–RC column, the BHTS buffer interlayer is capable of significantly diminishing the impact force of the RC column, with the impact force of the RC column being reduced by 77.89% on average, and the impact time history increased by 108.09% on average, which effectively alleviated the inertial force. In the context of the UHPC–BHTS–RC column configuration, it is evident that the UHPC–BHTS composite structure is capable of markedly diminishing the impact force exerted on the RC column and elongating the impact time history. The mean impact force of the RC column decreased by 66.21%, while the average impact time history was augmented by 95.07%.

### 3.3. Distribution of Displacement

**Figure 13:**
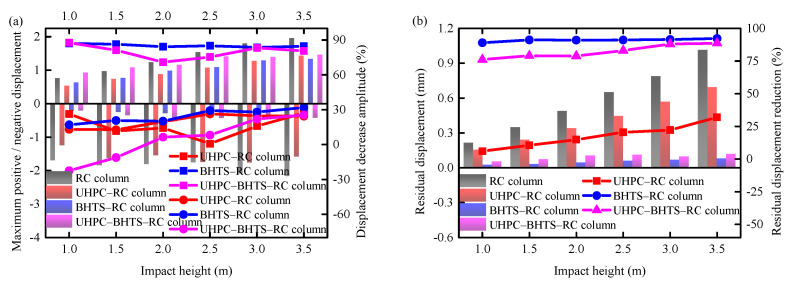
Comparison of displacement response: (**a**) maximum displacement; (**b**) residual displacement.

The maximal lateral and residual displacement of the RC column under each working condition of the three anti-collision devices are shown in Figure 13. It can be observed from Figure 13a,b that under the UHPC–RC condition, the mean values of the maximal and residual displacement were less than those of the RC column. It demonstrates that the UHPC anti-collision structure is capable of marginally diminishing the displacement response and residual displacement of the RC column. The RC column maximum positive/negative displacement decreased by 20.17%/15.97%, respectively, and the residual displacement was curtailed by 17.68% on average. The BHTS–RC column’s maximal displacement and the residual displacement were both less than those of the RC column lacking a protective device. As can be observed from Figure 13a,b, the BHTS buffer interlayer is capable of significantly diminishing the displacement response as well as the residual displacement of the RC column. On average, the maximal positive/negative displacement of the RC column decreased by 24.47%/85.11%, respectively, and the residual displacement was reduced by 91.00% on average. In the scenario of the UHPC–BHTS–RC column, the mean values of the maximal displacement and the residual displacement were smaller compared to those of the RC column without an anti-collision device. From Figure 13a,b, it can be observed that the UHPC–BHTS anti-collision structure is capable of diminishing the displacement response as well as the residual displacement of RC columns. On average, the maximum positive/negative displacement of RC columns decreased by 4.71%/79.80%, respectively, and the residual displacement was reduced by 82.26% on average. This indicates that the UHPC–BHTS anti-collision structure remarkably enhances the lateral stiffness of RC columns under impact loading conditions.

It is worth noting that the displacement under the falling condition at a height of 3.0 m was marginally less than the lateral positive and negative displacements in the falling condition at a height of 2.5 m. The reason for this lies in the fact that the UHPC protective layer had undergone overall failure under the working condition of 3.0 m, along with the exterior wall of the restraining device having assimilated a portion of the kinetic energy of the steel ball. The diminution of the lateral rigidity of the UHPC anti-collision structure gives rise to more prominent local plastic deformation of the steel ball within the BHTS, thereby enabling it to absorb a greater amount of energy. Under the 2.5 m working condition, the exterior wall of the restraining device within the UHPC anti-collision structure still maintained a certain lateral rigidity without conspicuous plastic deformation.

## 4. The Influence of Anti-Collision Device on the Failure Mode Transformation of RC Column

For the purpose of more conveniently investigating the material damage behavior and failure mode transformation of RC columns, a 20 m high impact condition involving steel balls under high energy input was devised.

### 4.1. Failure Phenomena

**Figure 14:**
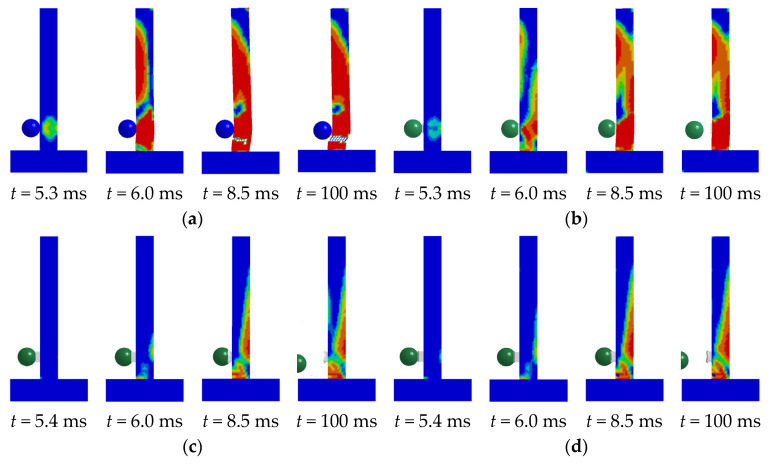
Development process of RC column concrete damage: (**a**) RC column; (**b**) UHPC–RC column; (**c**) BHTS–RC column; (**d**) UHPC–BHTS–RC column.

Figure 14a presents the progression of concrete damage within unprotected RC columns. The RC column experienced shear or punching failure under the combined influence of impact force and axial force. Figure 14b illustrates the evolution process of concrete failure within the context of a UHPC–RC column. The RC column experienced shear failure under the combined influence of the subsequent impact force and axial force. Figure 14c depicts the concrete failure evolution process of the BHTS–RC column. It can be discerned from the figure that the flexural damage of the RC column was situated at the bottom section of the column as well as the rear of the impact point. The BHTS–RC column underwent bending under the combined effect of impact force and axial force. Figure 14d represents the concrete failure evolution process of the UHPC–BHTS–RC column condition, and its development process bears a high degree of similarity to that of the BHTS–RC column condition.

### 4.2. Impact Force

**Figure 15:**
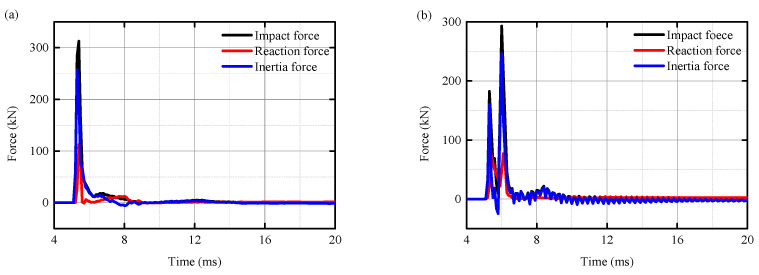

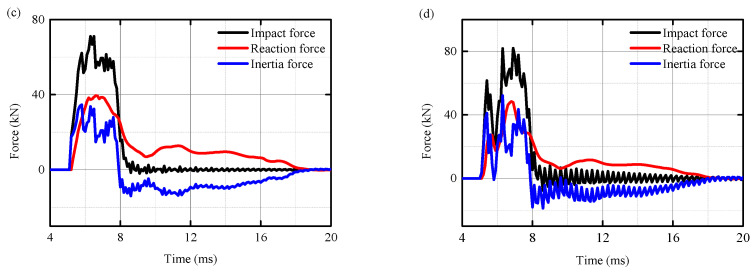
Dynamic time–history curve: (**a**) RC column; (**b**) UHPC–RC column; (**c**) BHTS–RC column; (**d**) UHPC–BHTS–RC column.

Figure 15 presents the time–history curves of the impact force, base horizontal reaction force, and inertial force of the RC column, UHPC–RC column, BHTS–RC column, and UHPC–BHTS–RC column, respectively. Comparing the shear failure in Figure 15a,b with the bending failure in Figure 15c,d, it can be seen that the bending–shear failure of the RC column exhibited a similar impact force time–history curve. It can be seen from Figure 15 that the impact force was larger when shear failure occurred in the two impact conditions without an anti-collision device and with a UHPC anti-collision structure, while the impact force in the impact conditions with the BHTS and UHPC–BHTS anti-collision structures was smaller. The three protective conditions (UHPC–RC column, BHTS–RC column, and UHPC–BHTS–RC column) reduced the impact force by 6.41%, 77.24%, and 74.03%, respectively, compared with the unprotected impact force. The bearing reaction force was reduced by 31.25%, 65.17%, and 57.14%, respectively. The inertial force decreased by 4.28%, 86.77%, and 79.76%, respectively. The duration of the pulse section increased by 16.67%, 183.33%, and 175.00%, respectively, demonstrating that the anti-collision device played an efficacious function in safeguarding the RC column.

### 4.3. Horizontal Displacement

**Figure 16:**
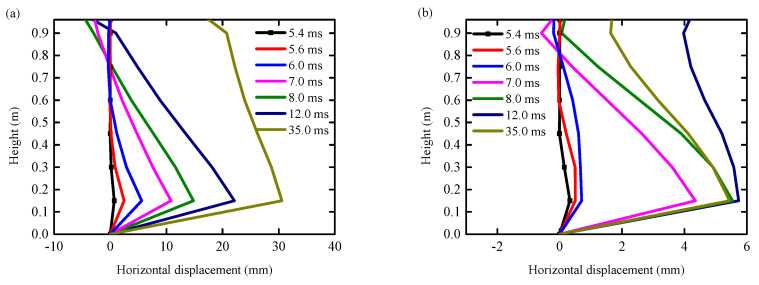

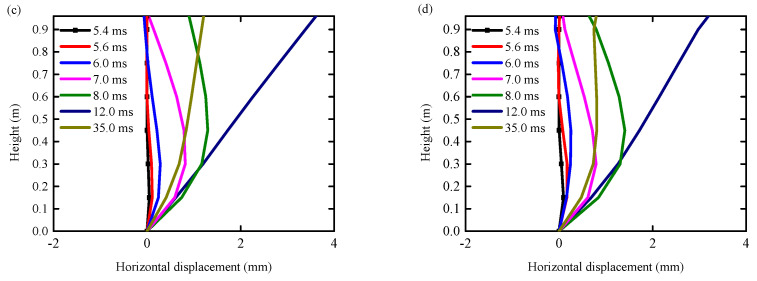
Horizontal displacement curves at different heights: (**a**) RC column; (**b**) UHPC–RC column; (**c**) BHTS–RC column; (**d**) UHPC–BHTS–RC column.

Figure 16a,b presents the horizontal displacement curve of each observation point on the central axis at a typical moment under the condition of the RC column and UHPC–RC column. It can be observed that the deformation of the unprotected RC column during the impact process can be categorized into two modes: local shear deformation and overall bending deformation. The primary factor contributing to the amplification of the overall flexural deformation is the establishment of plastic hinges at the base of the column. It can be discerned that during the course of shear failure, the local shear deformation of the RC column at the impact location (the initial phase of impact) swiftly transmuted into the overall flexural deformation and eventually developed into shear failure, predominantly governed by the local shear deformation at the impact position.

Figure 16c,d present the horizontal displacement curve of each observation point on the central axis at a typical moment under the condition of the BHTS–RC column and UHPC–BHTS–RC column. The displacement at the top of the RC column gradually augmented and began to surpass the horizontal displacement at the impact point. When *t* exceeded 40 ms, the overall bending deformation of the RC column was essentially restored.

### 4.4. Impact Kinetic Energy Transformation

**Figure 17:**
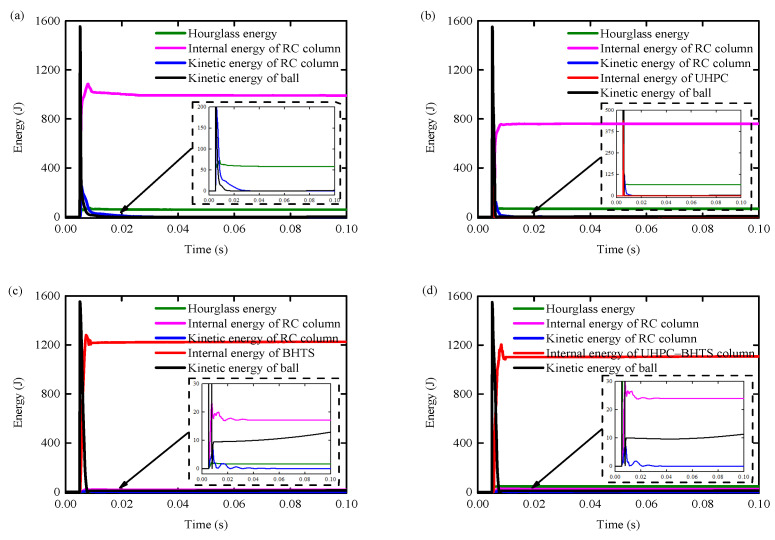
Time–history curve of energy transformation: (**a**) RC column; (**b**) UHPC–RC column; (**c**) BHTS–RC column; (**d**) UHPC–BHTS–RC column.

Figure 17a–d presents the temporal evolution curve of energy transformation during the impact process of the RC column, UHPC–RC column, BHTS–RC column, and UHPC–BHTS–RC column under the corresponding working conditions for each. The ratio of energy consumption regarding certain types of energy (such as slip energy, kinetic energy, and the internal energy of specific components) was not described. The distribution of energy alteration and total impact kinetic energy at each stage is illustrated in Table 2.

It can be seen from Table 2 that the protection of BHTS and UHPC–BHTS anti-collision devices absorbs most of the impact kinetic energy, and the RC column only absorbs a small part of the energy, so the RC column only has a bending failure, which has little effect on the overall structure. The RC column without protection and the UHPC anti-collision structure retain a large amount of kinetic energy after protection, so the remaining energy must be absorbed by the concrete and steel through energy dissipation, resulting in shear failure and the formation of plastic hinges (Figure 14a,b).

## 5. Damage Assessment Under Various Working Conditions

### 5.1. Dynamic Shear Capacity

Some researchers have conducted experimental and numerical investigations on the shearing mechanism of concrete structures subjected to impact loading [36,37,38]. The dynamic shear capacity ($V_{dsc}^{M}$) of the shear crack mode of the steel ball impacting the RC column within the aforementioned study can be formulated as [36]:

(1) $$V_{dsc}^{M}=DIF_{c}\times V_{c}+DIF_{s}\times V_{s}+\sum ma$$

Among them, $DIF_{c}$ and $DIF_{s}$ are the dynamic increase factors of concrete and steel strength on the inclined section; $V_{c}$ and $V_{s}$ are the static shear capacities of concrete and transverse reinforcement, respectively. *M* and a are the overall mass and acceleration of the shear zone, respectively.

The simplified calculation formula of the dynamic shear capacity is [36].

(2) $$V_{dsc}^{M}\approx \frac{0.3\times f_{c}\times W_{c}\times L}{\beta }=115.7\;kN$$

where $W_{c}$ is the width of RC column specimen; *L* is the calculated impact height, take 0.12 m; β is the shear span ratio of 1.

### 5.2. Peak Dynamic Shear Demand

The comprehensive damage assessment approach of bridge piers and entire bridge structures subjected to vehicle impact hinges on the ratio of the peak dynamic shear demand to the dynamic shear capacity of RCBP [36], that is, the damage index (*D*), which is expressed as follows:

(3) $$\lambda_{D}=\frac{V_{dsd}^{P}}{V_{dsc}^{M}}$$

Among them, $V_{dsd}^{P}$ and $V_{dsc}^{M}$ are the peak dynamic shear demand and dynamic shear capacity of RCBP, respectively. There is a linear correlation between $V_{dsd}^{P}$ and $F_{veh}^{P}$, which can be expressed by the following empirical formula [36]:

(4) $$V_{dsd}^{P}=0.65F_{veh}^{P}+200$$

where $F_{veh}^{P}$ is the peak collision force of the vehicle. In accordance with the scale factor of the concentrated force within the scale model amounting to 0.009, Equation (4) is rewritten as:

(5) $$V_{dsd}^{P^{\prime }}=0.65F_{veh}^{P}+1.8$$

### 5.3. Comparison of Pier Damage Assessment

Owing to the diverse impact forces exerted by vehicles, RC piers will experience varying degrees of damage, which will exert an influence on the subsequent service life of the piers subjected to impact [39,40]. D. Zhou and R. Li [41] improved the previous damage grade evaluation method and divided the damage into four grades: slight damage $D_{1}\;\left(0<\lambda \leq 0.4\right)$, meaning that the impacted pier can remain in service without repair; moderate damage $D_{2}\;\left(0.4<\lambda \leq 1.0\right)$, indicating that the piers should be repaired before continued use; severe damage $D_{3}\;\left(1.0<\lambda \leq 1.2\right)$, showing that a large lateral displacement is generated; and collapse $D_{4}\;\left(\lambda >1.2\right)$, indicating that the impacted pier is completely destroyed.

**Figure 18:**
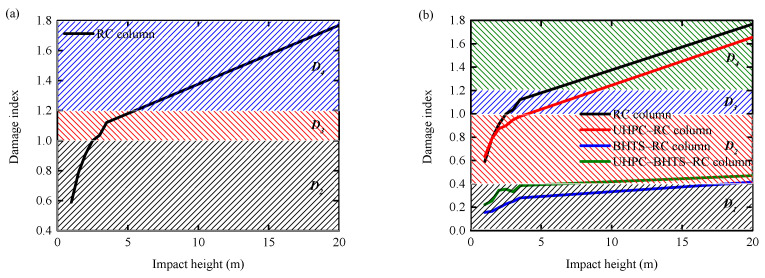
Damage assessment of RC columns: (**a**) without anti-collision structure; (**b**) with anti-collision structure.

The damage to RCBP and BHTS–RC columns is depicted in Figure 18. From the diagram, it can be seen that the RC column lacking the BHTS buffer interlayer has $D_{2}$ and $D_{3}$ zones, signifying that it has endured severe damage; the RC column equipped with the BHTS buffer interlayer is predominantly within the $D_{1}$ zone under the impact condition of the steel ball at a height ranging from 1.0–20 m, demonstrating that the BHTS buffer interlayer effectively safeguards the structural integrity of the RC column during impact.

## 6. Conclusions

In this paper, the protective effects of the UHPC anti-collision structure, the BHTS buffer interlayer, and the UHPC–BHTS anti-collision composite structures on RC columns subjected to impact loads are comprehensively investigated and contrasted through experiments and numerical simulations. The following conclusions can be drawn by obtaining and analyzing their influence on the impact force, displacement, energy absorption mechanism, and failure mode of RC columns:

1. By comparing the impact force, displacement, and other indicators, the impact force and residual displacement of the UHPC–RC column, BHTS–RC column, and UHPC–BHTS–RC column are reduced by 4.81%/77.89%/66.21% and 17.68%/91.00%/82.26%, respectively, compared to the RC column. The reason is that the protection of the BHTS and UHPC–BHTS anti-collision devices absorbs most of the impact kinetic energy, and the RC column only absorbs a small part of the energy, so the RC column only has bending failure, which has little effect on the overall structure. The RC column without protection and the UHPC anti-collision structure protection still retain a large amount of kinetic energy to be absorbed after protection, so it can only be absorbed by the concrete and steel through energy dissipation, thus forming shear failure. The BHTS buffer interlayer and UHPC–BHTS composite structure, with stronger protective performance, transform the RC column from the shear failure mode to the bending failure mode with reduced damage.
2. The damage to RC columns was evaluated. RC columns protected by the BHTS buffer interlayer and the UHPC–BHTS composite structure were in the $D_{1}$ and slight $D_{2}$ zones under the impact condition of steel balls at a 1.0–20 m height, while RC columns without anti-collision devices and with UHPC anti-collision structures appeared in the $D_{3}$ and $D_{4}$ zones, which were seriously damaged. This shows that the BHTS buffer interlayer and the UHPC–BHTS composite structure play a very effective protective role for RC columns, while the UHPC anti-collision structure has a limited protective effect on RC columns.
3. The effectiveness of the three anti-collision structures designed for the working conditions of this study is as follows: UHPC anti-collision structure < UHPC–BHTS composite structure < BHTS buffer interlayer. Under the impact energy of 20 m, the energy absorption performance of the three structures, along with the proportion of total kinetic energy, is as follows: 28.12%, 76.42%, and 82.33%, respectively. If the impact input energy continues to increase, the effectiveness of the three anti-collision structures is as follows: UHPC anti-collision structure < BHTS buffer interlayer < UHPC-BHTS composite structure, because the EA of the BHTS buffer interlayer has basically reached its limit, while the UHPC–BHTS composite structure still has a good EA reserve. In addition, considering that the durability of the UHPC–BHTS composite structure is better in practical engineering, the UHPC–BHTS composite structure is the most effective new protective structure among the three designed anti-collision structures.

## Figures and Tables

**Table 1:** Composition of UHPC.

Component	Portland Cement (kg)	Quartz Sand (kg)	Silica Fume (kg)	Nano-Calcium Carbonate (kg)	High Range Water Reducer (kg)	Water (kg)	Steel Fiber (%)
Quantity	750	1030	415	63.1	16	245.6	2

**Table 2:** Energy transformation in each stage.

Stage	Energy Type	I (%)	II (%)	III (%)	IV (%)	Total (%)
1	Kinetic energy of ball	1.1	21.78	34.46	42.45	99.79
	Internal energy of concrete	0.45	13.30	23.20	32.81	69.76
2	Kinetic energy of ball	1.23	18.70	14.59	65.10	99.62
	Internal energy of UHPC	0.89	16.90	10.33	/	28.12
	Internal energy of concrete	/	/	5.59	43.06	48.65
3	Kinetic energy of ball	34.54	12.63	26.67	25.32	99.16
	Internal energy of BHTS	30.36	11.35	22.13	18.49	82.33
4	Kinetic energy of ball	33.15	18.94	17.83	29.33	99.25
	Internal energy of UHPC-BHTS	26.49	13.53	11.73	24.67	76.42

## Data Availability

The original contributions presented in the study are included in the article, further inquiries can be directed to the corresponding authors. The raw data supporting the conclusions of this article will be made available by the authors on request.
